# Comparative Chloroplast Genomics of Endangered *Euphorbia* Species: Insights into Hotspot Divergence, Repetitive Sequence Variation, and Phylogeny

**DOI:** 10.3390/plants9020199

**Published:** 2020-02-05

**Authors:** Arif Khan, Sajjad Asaf, Abdul Latif Khan, Tariq Shehzad, Ahmed Al-Rawahi, Ahmed Al-Harrasi

**Affiliations:** 1 Natural and Medical Sciences Research Center, University of Nizwa, Nizwa 616, Oman; arif.biotec@gmail.com (A.K.); sajadasif2000@gmail.com (S.A.); Ahmed@unizwa.edu.om (A.A.-R.); 2 Genomics Group, Faculty of Biosciences and Aquaculture, Nord University, 8049 Bodø, Norway; 3 Department of Biological and Environmental Sciences, College of Arts and Sciences, Qatar University, 2713 Doha, Qatar; shehzad@uga.edu

**Keywords:** chloroplast genomics, genus *Euphoria*, comparative analyses, *Euphorbiaceae*

## Abstract

*Euphorbia* is one of the largest genera in the Euphorbiaceae family, comprising 2000 species possessing commercial, medicinal, and ornamental importance. However, there are very little data available on their molecular phylogeny and genomics, and uncertainties still exist at a taxonomic level. Herein, we sequence the complete chloroplast (cp) genomes of two species, *E. larica and E. smithii*, of the genus *Euphorbia* through next-generation sequencing and perform a comparative analysis with nine related genomes in the family. The results revealed that the cp genomes had similar quadripartite structure, gene content, and genome organization with previously reported genomes from the same family. The size of cp genomes ranged from 162,172 to 162,358 bp with 132 and 133 genes, 8 rRNAs, 39 tRNA in *E. smithii* and *E. larica*, respectively. The numbers of protein-coding genes were 85 and 86, with each containing 19 introns. The four-junction regions were studied and results reveal that *rps19* was present at J_LB_ (large single copy region and inverted repeat b junction) in *E. larica* where its complete presence was located in the IRb (inverted repeat b) region in *E. smithii*. The sequence comparison revealed that highly divergent regions in *rpoC1*, *rpocB*, *ycf3*, *clpP*, *petD*, *ycf1*, and *ndhF* of the cp genomes might provide better understanding of phylogenetic inferences in the *Euphorbiaceae* and order Malpighiales. Phylogenetic analyses of this study illustrate sister clades of *E. smithii* with *E. tricullii* and these species form a monophyletic clade with *E. larica*. The current study might help us to understand the genome architecture, genetic diversity among populations, and evolutionary depiction in the genera.

## 1. Introduction

Plants have chloroplasts (cp) that help in photosynthesis [1]. The genomic component of cp is composed of circular and double-stranded DNA molecules [2]. Moreover, it is very essential for fatty acids, starch, and pigments biosynthesis [3]. The chloroplast contains its own independent genomic component, which is highly conserved in angiosperms. The chloroplast genome possesses certain characteristics such as small single copies, multiple copies, and a simple structure [4]. Unlike the other genomes, such as the nuclear genome, which has more repetitive sequences, the mitochondrial genome in which frequent rearrangements of nucleotide occur, the chloroplast genome is conservative [5]. The chloroplast genome is maternally inherited in angiosperm, having its own independent evolutionary route [6]. The chloroplast genome shows collinearity among the plant kingdom, which is why phylogenetic trees are constructed on the basis of chloroplast data, and the genome structure of chloroplast provides information regarding the specie origin, evolution, and also the differences between closely related and other species [7]. In recent years, with the advent of advanced sequencing technology, more chloroplast genomes have been sequenced [4]. In this study, we sequenced the complete chloroplast genome of ecologically endangered species *E. smithii* and *E. larica* and performed a comparative analysis with other genomes from the Euphorbiaceae family.

The Euphorbiaceae (Spurge family) is one of the largest families in angiosperm and comprises 300 genera and almost 7500 species [8]. *Euphorbia larica* Boiss. and *Euphorbia smithii* S. Carter belong to the genus *Euphorbia*, which is the largest genus in the Euphorbiaceae family, comprising almost 2000 identified species, which mostly produce latex and possess a unique flower structure [9]. The genus is estimated to have originated in Africa approximately 48 million years ago and expanded to the American continents through two single long-distance dispersal events, i.e., 30 and 25 million years ago [10,11,12,13]. *Euphorbia* is an ecologically, medicinally, and commercially important genus in the Euphorbiaceae family, and various indigenous based traditional folk recipes are utilized as medicines for curing skin diseases, intestinal parasites, gonorrhea, warts, and migraines [14]. The *Euphorbia* species constitutes monocyclic diterpenoids that possess anti-bacterial, anti-cancer, anti-HCV, and analgesic activities [15,16,17]. Some plants of this genus secrete a sap which prevents the growth of other species and shows their habitat dominance feature [18]. In addition, some plants from this genus (for example, *E. pulcherrima*) is used for ornamental purposes [19,20]. 

In the case of *E. larica* and *E. smithii*, *E. larica* is native and widely found in northern regions of Oman [21], whilst *E. smithii* (near-threatened) was once considered endemic to Oman but has also been found in Yemen [22]. *E. larica* is a woody species with a self-supporting habitat, whereas *E. smithii* is a shrub [23]. The species are rich in flavonoids [23], alkaloids [9], and terpenoids [23]. The latex of *E. smithii* is used for veterinary medicines at a local level [24]. Similarly, the latex derived from *E. larica* is used to treat the camel parasite [25]. There are several examples of understanding the genetic diversity of the *Euphorbia* species, such as *E. telephioides* [26] and *E. pulcherrima* [27]. However, no study has been performed on *E. larica* and *E. smithii* due to the lack of genome or related sequence data. Understanding the genetic diversity is essential to ensure increased conservation efforts for the decline of such endemic or native species in the world. Looking at the importance of these species, we sequenced the complete chloroplast genome of two important species, *E. larica* and *E. smithii*, and performed comparative analysis with related species (*E. esula*, *E. tirucalli*, *M. esculanta*, *J. curcas*, *H. brasiliensis*, *R. communis*, *V. fordii*, *C. tiglium*, and *D. tonkinensis*). In our study, the sequencing of complete chloroplast genomes of *E. larica* and *E. smithii* encourages and provides a basis for a more detailed study of chloroplast molecular biology and also helps in the genetic breeding and molecular evolution of this threatened species. This study also provides details of evolutionary analysis and helps in the classification of this morphologically diverse species. Some previous studies have suggested that this group has been difficult to discern mainly due to homoplasious morphological characters and inadequate taxon sampling in previous phylogenetic studies [28].

## 2. Results 

### 2.1. Comparative Characteristics of Chloroplast genomes

**Figure 1:**
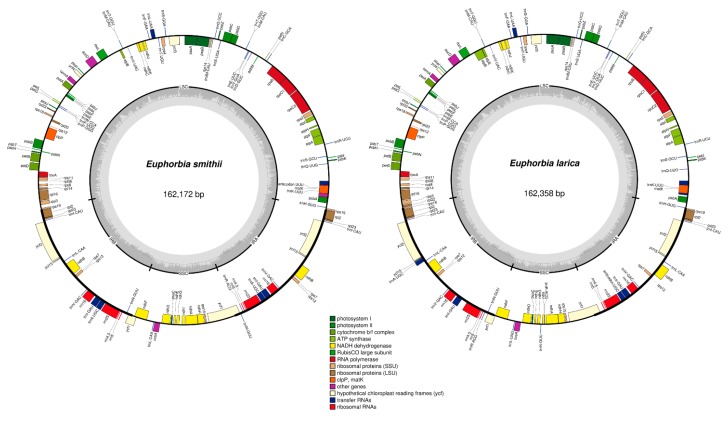
Genome circular map of the *E. smithii* and *E. larica*. Thick lines indicate the extent of the inverted repeat regions (IRa and IRb), which separate the genome into small (SSC) and large (LSC) single copy regions. Genes drawn inside the circle are transcribed clockwise, while those outside of the circle are transcribed counter-clockwise. Genes belonging to different functional groups are color-coded. The dark gray in the inner circle corresponds to the GC content, while the light gray corresponds to the AT content.

The chloroplast genome of the *Euphoria* species showed a typical tetrad quadripartite structure comprising (i) small single copy, (ii) large single copy, (iii) inverted repeat A, and (iv) inverted repeat B that are mirror images of each other (Figure 1A,B). The complete chloroplast genome of *E. smithii* was 162,172 bp, which is 186 bp less than *E. larica* (162,358 bp). The two sequenced genomes were compared with two from the *Euphorbia* genus (*E. esula and E. tirucalli*) and seven (*M. esculanta*, *J. curcas*, *H. brasiliensis*, *R. communis*, *V. fordii*, *C. tiglium*, and *D. tonkinensis*) other cp genomes from the Euphorbiaceae family. The large single-copy region (LSC) of *E. smithii* was observed with a length of 91,158, while the LSC of *E. larica* was 91,537 bp in length. The length of the small single copy region of *E. smithii* (18,603 bp) was 364 bp larger than *E. larica* (18,239 bp). The smallest IR region length was 10,100 bp in *C. tiglium* and the highest was observed in the *R. communis* (27,347 bp). Overall, there were little differences among the two sequenced genomes, and the main differences were in the LSC and SSC regions. The complete chloroplast genome ranges from 150,021 bp in *C. tiglium* to 163,856 bp in *J. curcas* (Table 1).

The total numbers of genes annotated in *E. smithii* and *E. larica* were 132 and 133, respectively, including 85 and 86 protein coding genes (PCGs), 8 rRNAs, 39 tRNAs, and 19 intron-containing genes (Table 1). The number of protein-coding genes varied among these genomes, the highest of which were recorded in *E. larica* and *R. communis*, whilst the lowest were 78 in *C. tiglium*. The highest number of introns containing genes among the compared genomes was 21, noted in *J. curcas*, *H. brasiliensis*, and *V. fordii*, and the lowest were 13 in *E. tirucalli*. GC content of *E. smithii* was found higher than GC content of *E. larica*; the highest GC content found in the compared genome was 36% found in *V. fordii*, while the lowest was observed in *J. curcas* and *C. tiglium* with 35.4%. Relative conservation of the genome structure and gene contents were observed among all the eleven chloroplast genomes with no specific gene organization and rearrangement observed, though some differences were still found in the number of genes, intron losses, and contraction and expansion in the IR regions. The chloroplast genome contains some of the important genes responsible for the vital process of life, i.e., photosynthesis and self-replication of chloroplast being a self-replicating organelle in the plant cell (Supplementary Table S1). The self-replication of chloroplast includes the gene responsible for the large subunit of ribosomal proteins, the small subunit of ribosome, DNA dependent RNA polymerase, rRNA genes, and tRNA genes. The genes responsible for photosynthesis further include photosystem I and II, and 33 genes are responsible for carrying out photosynthesis in the chloroplast genome (Supplementary Table S1). 

The chloroplast genome of *E. smithii*, *E. larica*, *E. esula*, and *E. tirucalli* contains 18 introns containing genes. Out of these 18 introns containing genes, five are tRNAs, and three genes, *ycf3*, *clpP*, and *rps12*, contain double introns (Table 2). The base composition analyzed in sequenced and compared genomes reveal that adenine (A) at the first position was 31.0%, 31.2%, 31.0%, and 30.9% and on second position 29.7%, 29.8%, 29.8%, and 29.9%, while at third position 13.8%, 32.1%, 28.4%, and 32.4% in the *E. larica*, *E. smithii*, and *E. tirucalli*, respectively. Likewise, the composition of base T (thymine) at the first position was 24.3%, 25.9%, 24.1%, and 23.9%; at second position 32.6%, 32.7%, 28.4%, and 38.4%; and at third position 38.2%, 38.2%, 38.6%, and 38.4%, respectively. Furthermore, the abundance of “G” and “C” at the first, second, and third positions were observed less than the abundance of “A” and “T” (Table 3).

### 2.2. Analysis of Repetitive Sequences in the Genomes

**Figure 2:**
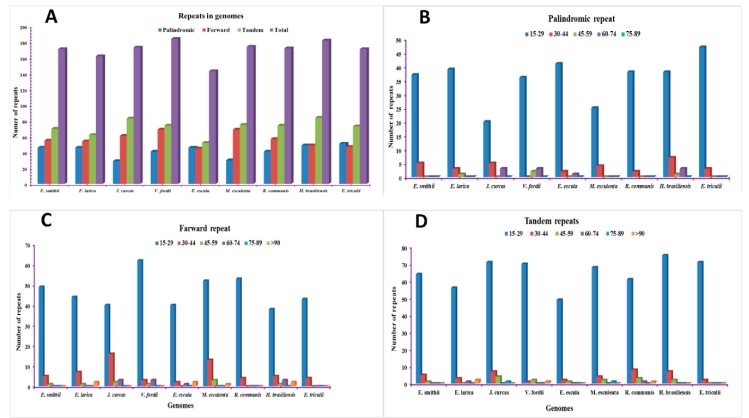
Analysis of repetitive sequences in *E. smithii* and *E. larica.* (**A**) Total number of repeats present in the genome. (**B**) Number of palindromic repeats in the genome. (**C**) Number of forward repeats present in the genome. (**D**) Number of tandem repeats present in the genome.

Repeat analyses of the two sequenced *Euphoria* species and seven other chloroplast genomes were conducted. The result of the repeats shows the total number of repeats present in *E. smithii* (171) and *E. larica* (162). Among the compared genomes, the highest number of repeats was found in *V. fordii* with 184, followed by *H. brasilliensis* with 182. Furthermore, the lowest repeats were found in *E. esula*, comprising 143 repeats (Figure 2). Among the repeats, the tandem repeats were found highest, followed by the forward and palindromic repeats. The repeats of different sizes were also studied. In *E. smithii*, the palindromic repeats were found to be 46 among which the 15–29 size repeats were found to be 37, and the 30–44 size repeats, as well as >90, were 4. The forward repeats were 55 in which 15–29 were found to be 49, and 30–44 were found to be 5. Among the 70 tandem repeats, 15–29 were 64, 30–44 were 5, 45–59 was 1. In *E. larica*, among the 46 palindromic repeats, the 15–29 were 39, 30–44 were 3, 45–59 were 1 and >90 were 3. The forward repeats were 54, among the 15–29 were 44, 30–44 were 7, 45–59 were 1, and >90 were 2. The tandem repeats were analyzed to be 62 in which 15–29 were 56, 30–44 were 3, 60–74 were 1, and >90 were 2 (Figure 2D). 

### 2.3. SSRs Polymorphism Analysis

**Figure 3:**
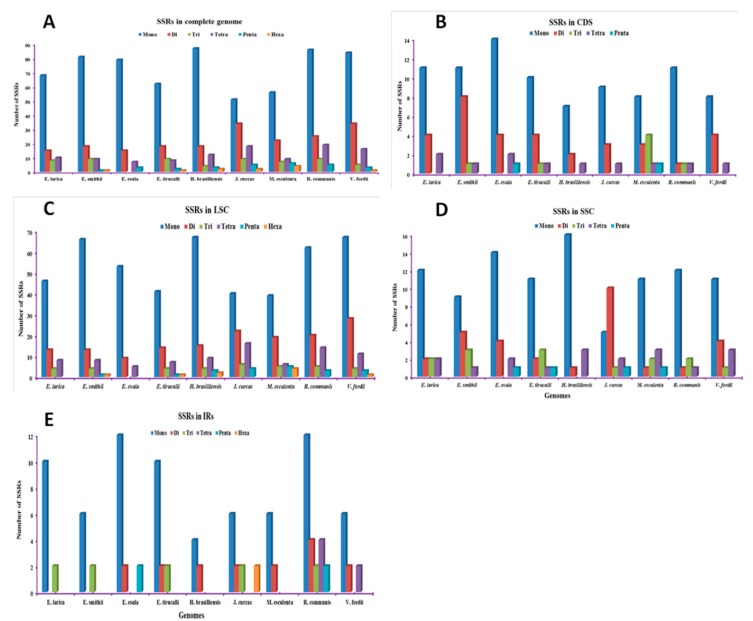
Analysis of simple sequence repeats (SSRs) in chloroplast genomes of *E. smithii* and *E. larica*. (**A**) Total number of SSRs present in complete genomes. (**B**) Total number of SSRs present in the CDS of the genome. (**C**) Total number of SSRs present in the LSC of genome. (**D**) Total number of SSRs present in SSC of the genome. (**E**) Total number of SSRs present in IRs of the genome.

Simple sequence repeats (SSRs) are the microsatellites present in the chloroplast genomes, which play an important role in the cp genome. They are usually varying from one to six base pairs and present in all genome. In our current study, we determined the SSRs in the sequenced and compared cp genomes. Our result for SSRs analysis reveals that there are 101, 119,104, 100, 126, 119, 104, 144, and 143 SSRs found in the *E. larica*, *E. smithii*, *E. esula*, *E. tirucalli*, *H. brasilliensis*, *J. curcas, M. esculenta*, *R. communis*, and *V. fordii* genomes, respectively (Figure 3A). The highest number of SSRs was found in *R. communis*, while the lowest was observed in *E. larica*. Moreover, the mono nucleotide in *E. larica* and *E. smithii* were highest with 68 and 81, while the lowest were tri in *E. larica* and hexa in *E. smithii*, which were found and were observed to be absent in the *E. larica* cp genome (Figure 3B). The SSRs present in the CDS region were 17 and 21, LSC region comprised 71 and 93, SSC region comprised an equal number, which is 18. In In inverted repeat regions, the number of SSRs was 12 and 8 in the *E. larica* and *E. smithii*, respectively (Figure 3C–E). In both *E. larica* and *E. smithii*, most mononucleotide SSRs were T (61.7%, 46.1%) motifs, with the majority of dinucleotide SSRs being A/T (8, 13) motifs (Supplementary Figure S1).

### 2.4. Compression and Augmentation of IR Region

**Figure 4:**
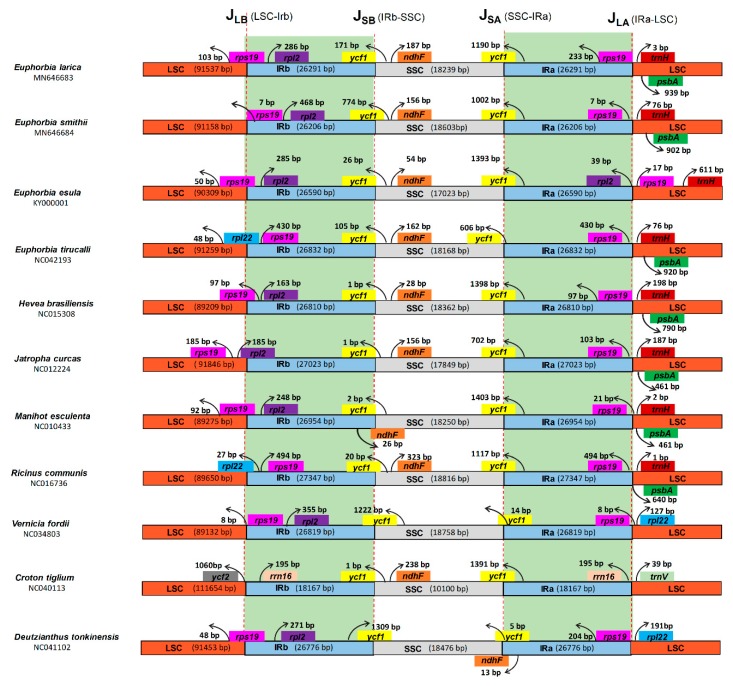
Distances between adjacent genes and junctions of the small single-copy (SSC), large single-copy (LSC), and two inverted repeat (IR) regions among plastid genomes *E. smithii* and *E. larica* and related species within the Euphorbiaceae family. Boxes above and below the primary line indicate the adjacent border genes. The figure is not to scale with regards to sequence length and only shows relative changes at or near the IR/SC borders.

The expansion and contraction of the inverted repeats at the border region in the chloroplast genome is commonly observed and mainly responsible for the size variation in the chloroplast genome. Therefore, for the complete study of inverted repeat regions in the sequenced genomes of *E. larica* and *E. smithii*, we compared the IR border regions and the genes present within these junctions with the other nine chloroplasts genomes. Critically analyzing the junctions of *E. larica* revealed that the length of LSC was found to be 91,537 bp and the *rps19* gene was located on the junction of LSC/IRb (J_LB_). The gene *rpl2* was located 286 bp from the J_LB_ in the IRb region. The *ycf1* gene was located at J_SB_ and J_SA_ junctions while the *ndhF* gene was located in the SSC region, 187 bp away from the J_SB_ junction. The J_LA_ junction includes the *rps19* gene and the *trnH* gene located in the IRa and LSC region, respectively. The *E. smithii* junctions contained the same genes present in the *E. larica*, with small differences in the location from the junction region like the *rps 19* gene that is located in the IRb region 7 bp away from the J_LB_ region. In all the compared genomes, the location of *rps19* at the J_LB_ junction shows a similar pattern in *E. larica*, *E. esula*, *H. brasilliensis*, *M. esulenta*, and *D. tonkinesis*, while the complete location of *rps19* in the IRb region was observed in the *E. smithii*, *E. tirucalli*, *R. communis*, and *V. fordii*. Surprisingly, the *rps19* was found completely in the LSC region in *J. curcas*. The *rps19* gene, like *ycf1*, is present at two locations in the junction region, while in some genomes, among the compared genome like *C. tiglium*, it was found completely absent at J_LB_, while at J_LA_ it was present in the IRa region (Figure 4).

The *ycf1* gene was present at both the J_SB_ and J_SA_ junction in all compared genomes. The *ndhF* gene was found in all compared genomes at the SSC region near the J_SB_ junction, except the few genomes like in *M. esulenta* and *D. tonkinensis*. It is present at the J_SB_ while absent in the *V. fordii* genome. The *trnH* gene was present in the LSC region near the J_LA_ junction in all genomes, except *V. fordii*, *C. tiglium*, and *D. tonkinesis*, where it was missing and was replaced by *rpl22*, *trnV*, and *rpl22* in these genomes, respectively. Surprisingly, the *rrn16* gene was found in the IRa and IRb regions. The *C. tiglium* genome was absent in all other sequenced and compared genomes (Figure 4).

### 2.5. Comparison of the Hotspot Region in the cp Genome

**Figure 5:**
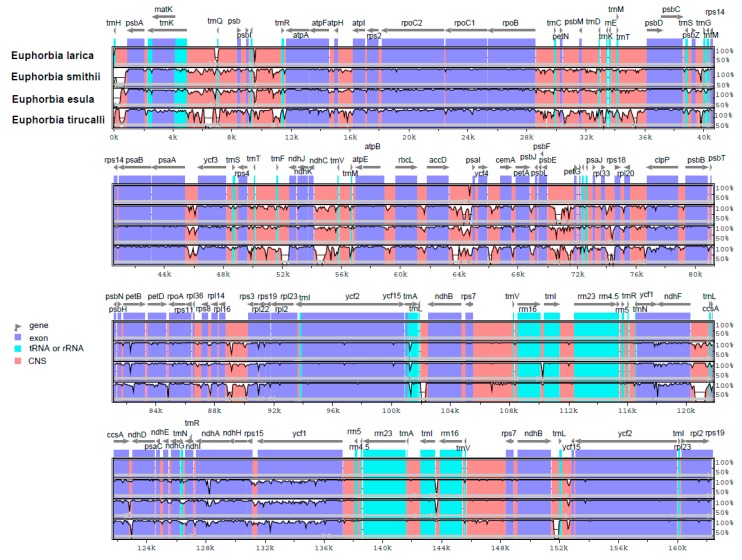
Visual alignment of plastid genomes with the previously reported cp genomes. VISTA-based identity plot showing sequence identities among eight species, using *E. larica* as a reference.

Chloroplast genomes present in most of the higher plants are relatively conserved and stable in terms of their structure and gene content. Despite the conserved structure, some variation in plant groups like genome size, gene content, and genome structure still occur due to the different evolutionary histories and genetic backgrounds. The *E. larica* cp genome was taken as a reference for detecting a divergence hotspot in *E. smithii*, *E. esula*, and *E. tirucalli*. The divergence in protein-coding genes was also analyzed and 65 genes were studied for the pairwise distance among these genomes. Sequence divergence analysis of the *Euphoria* species and compared genomes revealed a high conservative degree of the coding region as compared to non-coding regions. Furthermore, it was found that sequence divergence in the single copy region was higher than in inverted repeats regions. Further analysis of genes revealed that some of the divergent regions in these genomes were *rpoC1*, *rpocB*, *ycf3*, *clpP*, *petD*, *ycf1*, and *ndhF*. These regions were divergent but less divergent than non-coding regions (Figure 5).

### 2.6. Phylogenomic Analysis of E. larica and E. smithii and Its Comparison with Related Species

**Figure 6:**
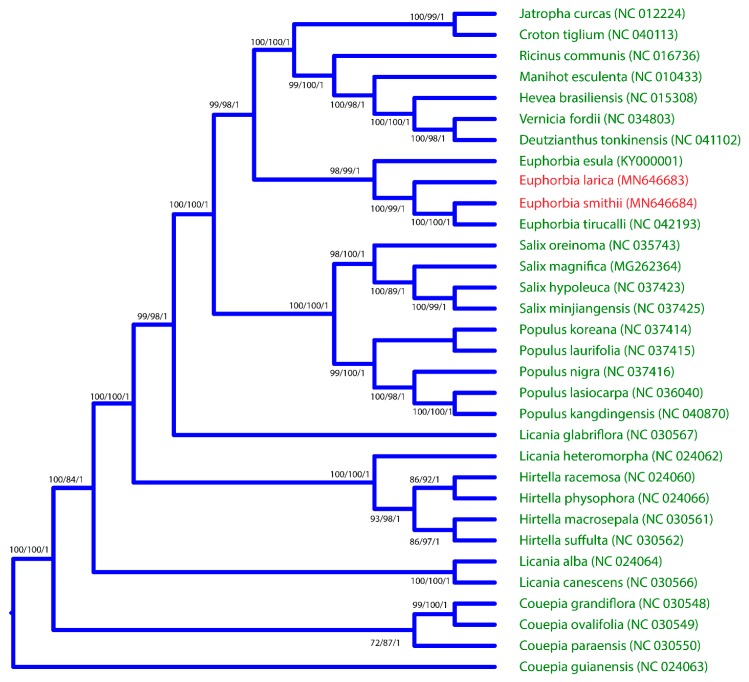
Phylogenetic trees of *E. smithii* and *E. larica*. The entire genome dataset was analyzed using three different methods: Bayesian inference (BI), maximum parsimony (MP), and maximum likelihood (ML). Numbers above the branches represent bootstrap values in the ML and MP, and posterior probabilities in the BI trees. Red color represents the positions of *E. smithii* and *E. larica.*

In this study, a dataset of 32 complete chloroplast genomes was used to construct the phylogenetic tree of *E. larica* and *E. smithii.* The *Couepia paraensis* chloroplast genome was used as an out group in this study. The phylogenetic tree was constructed using MP (maximum parsimony), ML (maximum likelihood), and BI (Bayesian interference). The result of the phylogenetic tree based on the complete chloroplast genome shows that *E. smithii* and *E*. *larica* share the same clade, which further makes a sister clade with *E*. *esula* with high bootstrap values (Figure 6). 

## 3. Discussion

With the advancement of next-generation sequencing technologies, the number of sequenced genomes has increased rapidly in the NCBI database. The availability of this data provides new insight into the chloroplast genomics, phylogenetic studies, rearrangement of genomes, sequence divergence, simple sequence repeats analysis, and the study of nucleotide substitution in these genomes. Euphorbiaceae is a large family and the number of the sequenced chloroplast genome is very limited [29]. The two sequenced cp genomes are comparatively analyzed with another cp genome to study the various parameters of these genomes. The chloroplast genome structure and gene order of these two chloroplast genomes are highly conserved with no specific genome inversion reported, and the gene order was the same and found consistent with previously reported genomes [30]. In the present study, we compared eleven chloroplast genomes. All of them were assembled into a single chloroplast genome presenting a typical quadripartite structure. Analysis of two sequenced *Euphorbia* genomes revealed that, like most of the higher angiosperm genomes, they comprised the tetrahedral structure containing two pairs of inverted repeats, one large single-copy region and one small single-copy region [4,31]. There was a 186 bp difference observed between the two sequenced chloroplast genomes, and the size was also comparable with other compared *Euphorbia* species, as well as the *Vernicia fordii* chloroplast genome, which is 161,528 bp in length [32], suggesting that chloroplast genomes are conserved. The result was consistent with previously reported studies [30]. The total number of genes presented in the chloroplast genome is divided into three main categories. The first is related to chloroplast gene expression and its self-replication. This includes the majority of rRNA, tRNA, and genes for RNA polymerase synthesis. The second category of genes is related to the vital process of life, i.e., genes responsible for photosynthesis, which includes photosystem I and photosynthesis II. The third category of genes is responsible for other biosynthesis genes and some genes of unknown function, such as *matK* and *ycf1* [33,34], similar to sequenced *Euphorbia* chloroplast genomes. During evolution, some genomes are liable to gain or lose introns, and this process plays a key role in expression and gene regulation [35]. In the *Euphorbia* species, there are 12 genes and 6 tRNA which contain introns and were found to be similar in the previously reported *V. fordii* cp genome belonging to Euphorbiaceae [32]. Some of the genes in the chloroplast genome contain double introns, such as *rps12*, *clpP*, and *ycf3*, and in some genes like *rpl2* and *rpl16*, the second intron is absent, which is consistent with previously reported genomes of *Manihot esculenta* [35] and *Oresitrophe* [36]. This phenomenon was absent in *H*. *micrantha* cp genomes (EF207446). The GC content of these sequenced genomes was consistent with a previously reported genome from this genus [37,38]. The number of repeats, including forward, tandem, and palindromic repeats, were studied in the chloroplast genomes sequenced and compared, and were found in a larger amount than in the previously reported cp genome of *V. fordii* (49 repeats) [32]. Among these repeats, tandem repeats were found several times more than palindromic and forward repeats, which are consistent with the *Teucrium* and *Commiphora* species [30,31], as well as *S. miltiorrhiza* [39], as previously reported. 

SSRs (simple sequence repeats) are repeats that play an important role in genome stabilization and rearrangement of genome sequences, and these SSRs make the cp genome favorable because of its use as a molecular maker and phylogenetic analysis [40,41]. In our study 101 and 119 total SSRs were found in *E. larica* and *E. smithii* respectively, which is higher than the Euphorbiaceae family members [32]. However, it is similar to the previously reported *B. sacra* cp genome [42]. Among the dinucleotide SSRs, AT was found to be the most abundant in the sequenced and compared cp genomes, similar to the previously reported genomes [30]. Another and important characteristic of the chloroplast genome, which is useful for evolutionary studies, is the location of the boundaries among the four chloroplast regions. Evaluating their contraction and expansion can shed some light on the evolution of some taxa [43]. From our results, we noticed that the length variation in the IR regions created some pseudogenes, like the *ycf1^Ψ^* or *rps19^Ψ^*. The *ycf1* pseudogene is present in all studied species, whereas the *rps19* pseudogene is only present in *C*. *icaco*, *H*. *racemose* (*Chrysobalanaceae*), *V*. *seoulensis* (*Violaceae*) [44], and *M*. *esculenta* (*Euphorbia*ceae) [35]. Inverted repeats are the most conserved region in the chloroplast genome and the construction and expansion of these IR regions are the common evolutionary events that lead to the differences in the size of chloroplast genomes [45]. In most of the plants, the border and junctions of the quadripartite structure of the genome structure is conserved but some species show inversion at the junction, as previously reported in [46], and loss of genes reported in [47], as well as contraction and expansion, which is a common event observed in the cp genomes of angiosperms [48]. Some angiosperm also show the loss of inverted repeats, such as geranium [49] and *fabaceae* [50]. Our study analysis of junction regions shows that the *rps19* gene is present at the J_LB_ junction in *E. larica*, while other genes like *ndhF* also show a pattern that is similar in *Violaceae*, as previously reported [44]. The *rps19* gene present in the IRb region near the J_LB_ junction was found in the present study of the *Byrsonima* species reported by Alison et al. [51]. Previously, it was identified that the alignment of many genomes contributes and identifies mutational hotspots, which are widely used for interspecies discrimination and species-level phylogenetic studies [52]. The coding region in many previous studies has been proven to play an important role in species-level phylogenetic analyses like some of the genes, such as *ycf1* in *Anemopaegma* [53] and *rps16*, *psaI*, *psbT*, *psbH*, *petB*, *rpoA*, and *rps11* in *Notopterygium* [54], which were more divergent than non-coding regions. However, a number of studies have confirmed that there is more variation in the non-coding region comprising the intergenic spacer regions and introns. For species identification in some previous studies, the *clpP*, *rps16*, *rpoB-trnC*, *rbcL-accD*, and *ccsA-ndhD* regions were used as markers [55] and *trnH-psbA*, *trnG-trnM*, *trnT-trnL*, *rpl32-trnL*, *rps15-ycf1*, *ycf4-cemA*, and *petD-rpoA* were the divergence hotspot regions in *Veroniceae* and *Veronica* [48]. 

In our study, the four *Euphorbia* species were compared through mVISTA and multiple alignment analyses. It was revealed that some of the regions were found more divergent and consisted of non-coding regions as compared to coding regions. Some regions, like *rpoC1*, *rpocB*, *ycf3*, *clpP*, *petD*, *ycf1*, and *ndhF*, were larger in number. These results are consistent with previously reported cp genomes [23,28]. Furthermore, we screened the four most mutational hotspots, *ndhF*, *ycf1*, *ndhA*, and *rpl32-trnL*, which can be used as genetic markers for species delimitation and phylogenetic studies of the genus *Euphorbia*. However, our study finds that more hotspot regions were present in the SSC region while the IR region was conserved, similar to the previously reported [56]. The phylogenetic position of genus *euphorbia* and our sequenced species were not identified on the basis of the complete chloroplast genome. Previously, some phylogenetic study was carried out on the basis of ITS regions and the plastid *ndhF* gene [57]. Based on the previous studies, it was not possible to understand the position of these two sequenced *E. smithii* and *E. larica* and the compared genome in this genus. Our study, on basis of complete cp genome sequences, provides a detail of the phylogenetic position of genus *Euphorbia* species. The current study reported for the first time sequence datasets of the two species, and it might help us to understand the genome architecture, genetic diversity amongst populations, and evolutionary depiction in the genera. 

## 4. Material and Methods

### 4.1. Chloroplast DNA Extraction and Sequencing

Young fresh healthy green leaves of *E. larica* and *E. smithii* were collected from the Nizwa governorate (57°31′59.99″ E) and placed immediately in liquid nitrogen. The contamination-free chloroplast DNA was extracted according to a modified protocol of Shi et al. [58]. An ion torrent sequencing platform was used for the sequencing of these samples using the Ion Torrent S5 sequencer with an ion torrent server (Life Technologies, Carlsbad, CA, USA). Genomic libraries were prepared according to the manufacturer’s instructions (Life Technologies, Carlsbad, CA, USA). Total chloroplast DNA of each sample was sheared enzymatically for 400 bp using the Ion Shear™ Plus Reagents kit, and libraries were prepared using the Ion Xpress™ Plus gDNA Fragment Library kit. Prepared libraries were quantified and qualified on a Qubit 3.0 fluorometer and bioanalyzer (Agilent 2100 Bioanalyzer system, Palo Alto, CA, USA). Libraries preparation was followed by template amplification (Ion OneTouch 2 instrument, Life Technologies, Carlsbad, CA, USA) and enrichment of the amplified template (Ion OneTouch™ ES enrichment system, Life Technologies, Carlsbad, CA, USA) by using Ion 520 and 530 OT2 reagents. The sample was loaded onto the Ion S5 sequencing chip and sequencing was performed according to the protocol of Ion Torrent S5 (Life Technologies, Carlsbad, CA, USA). 

### 4.2. Genome Assembly 

A total of 1,018,614 and 1,396,422 raw reads were generated for *E. larica* and *E. smithii*, respectively. The obtained reads of the genomes were mapped to the selected reference genome of *E. esula* using Bowtie2 (v.2.2.3) [59] in Geneious Pro (v.10.2.3) [60] software. The mean coverage of the assemblies for *E. larica* and *E. smithii* were 186X and 256X, respectively. The IR junction regions were identified using the already published genome of *E. esula*, and an iteration method using the MITObim (v.1.8) software [61] was utilized to adjust the sequence length. After sequencing, FastQC (v0.11.6) [61] was performed to check the read quality. To reduce biases in the analysis, an in-house script was used to filter out reads if less than 90% of the bases that made up the read were below Q20. Trimmomatic (v0.36) [62] was used to remove adapter sequences. Only high-quality reads were mapped using Bowtie2 in Geneious Pro (v.10.2.3) [60] as previously performed in cp genome of *Vachellia nilotica* [63].

### 4.3. Genome Annotation

Chloroplast genomes were annotated by using Dual Organellar Genome Annotator (DOGMA) [64], and BLASTX and BLASTN were used to identify the positions of ribosomal RNAs, transfer RNAs, and coding genes. The tRNAscan-SE version 1.21 [65] software was used to annotate tRNA genes. Additionally, for manual adjustment, Geneious and tRNAscan-SE [65] were used to compare it with previously reported genomes. Correspondingly, the start and stop codon and intron boundaries were also manually adjusted compared with a pre-published *E. esula* cp genome. In addition, the structural features of both *Euphorbia* species cp genomes were illustrated using OGDRAW [66]. Correspondingly, MEGA6 software [67] was used to determine the relative synonymous codon usage and deviations in synonymous codon usage by avoiding the influence of amino acid composition. The divergence of these three *Euphorbia* species taxa genomes with other related species (Figure 5) was determined by using mVISTA [68] in Shuffle-LAGAN mode, using *E. esula* as a reference genome.

### 4.4. Repeat Identification

REPuter software [69] was used for the identification of palindromic, forward, and tandem repeats present in the genome. The criterion was a minimum of >15 base pairs with a sequence identity of 90%. Furthermore, SSRs were determined using Phobos version 3.3.12 [70] with the search parameters set for mononucleotide repeats at ≥10 repeat units, for dinucleotide repeats at ≥ 8repeat units, for tri nucleotide and tetra nucleotide repeats at ≥4 repeat units, and for penta nucleotide and hexa nucleotide repeats at ≥3 repeat units. Tandem Repeats Finder version 4.07 b [71] with default settings was used to determine tandem repeats.

### 4.5. Sequence Divergence and Phylogenetic Analysis

The average pairwise sequence divergence of the complete cp genomes of *Euphorbia* species with related species was determined. Comparative sequence analysis after comparing gene order and multiple sequence alignment was used to identify missing and ambiguous gene annotations. MAFFT version 7.222 [72] with default parameters was used for the alignment of complete genomes, and pairwise sequence divergence was calculated by selected Kimura’s two-parameter (K2P) model [73]. To resolve the phylogenetic position of *E. larica* and *E. smithii* within the Euphorbiaceae family, cp genomes were downloaded from the NCBI database. Alignment of the complete cp genomes was constructed on the basis of conserved gene order and structure of the cp genome, and three different methods were applied to infer phylogenetic analysis: Bayesian inference (BI), implemented using Mr Bayes 3.1.2 [74,75]; maximum parsimony (MP), implemented using PAUP 4.0 [76]; and both maximum likelihood (ML), implemented using MEGA 6 [60], employing previously described settings [77,78]. For ML analysis, parameters were adjusted with a BIONJ tree with 1000 bootstrap replicates using the Kimura 2-parameter model with gamma-distributed rate heterogeneity and invariant sites. A heuristic search for MP analysis was run with 1000 random addition sequence replicates with the tree-bisection-reconnection (TBR) branch-swapping tree search criterion. The best substitution model, GTR + G model, was used according to the Akaike information criterion (AIC) by jModelTest version 2102 for Bayesian posterior probabilities (PP) in the BI analyses. The Markov Chain Monte Carlo (MCMC) method was run with four incrementally heated chains for 1,000,000 generations, starting from random trees, and sampling one out of every 100 generations. The first 25% of trees were discarded as burn-in to estimate the value of posterior probabilities.

## Figures and Tables

**Table 1:** Summary of complete chloroplast genomes of *E. laica* and *E. smithii.*

	*E. smithii*	*E. larica*	*E. esula*	*E. tirucalli*	*M. esculanta*	*J. curcas*	*H. brasiliensis*	*R. communis*	*V. fordii*	*C. tiglium*	*D. tonkinensis*
**Size (bp)**	162,172	162,358	160,512	163,091	161,453	163,856	161,191	163,161	161,528	150,021	163,481
**Overall GC contents**	35.8	35.6	35.6	35.6	35.9	35.4	35.7	35.7	36.0	35.4	35.7
**LSC size in bp**	91,158	91,537	90,309	91,259	89,275	91,846	89,209	89,650	89,132	111,654	91,453
**SSC size in bp**	18,603	18,239	17,023	18,168	18,250	17,849	18,362	18,816	18,758	18,167	18,476
**IR size in bp**	26,206	26,291	26,590	26,832	26,954	27,023	26,810	27,347	26,819	10,100	26,776
**Protein coding regions size in bp**	79,173	80,458	80,274	74,289	72,108	79,206	78,852	79,494	80,283	68,601	79,857
**tRNA size in bp**	2885	2887	2925	2885	2814	2797	2812	2802	2742	2560	2740
**rRNA size in bp**	9050	9049	9049	9049	6252	9047	9050	9050	9048	9050	9050
**Number of genes**	132	133	132	130	128	129	129	131	129	122	134
**Number of protein coding genes**	85	86	85	82	83	84	84	86	85	78	84
**Number of rRNA**	8	8	8	8	7	8	8	8	8	8	8
**Number of tRNA**	39	39	39	39	38	37	37	37	36	34	36
**Genes with introns**	19	19	18	13	20	21	21	19	21	20	20

**Table 2:** The genes with introns in the *Euphorbia* species chloroplast genome and the length of exons and introns.

Gene	Exon I (bp)				Intron 1 (bp)				Exon II (bp)				Intron II (bp)				Exon III (bp)			
	E.l	E.s	E.e	E.t	E.l	E.s	E.e	E.t	E.l	E.s	E.e	E.t	E.l	E.s	E.e	E.t	E.l	E.s	E.e	E.t
*atpF*	145	145	145	145	670	671	666	667	470	470	470	470								
*petB*	6	6			773	779			642	642										
*PetD*	8	8			779	780			496	496										
rpl2*	400	396	396	396	634	629	624	629	464	468	468	468								
*rpl16*	9	9			1395	1395			399	399										
*rpoC1*	430	432	432		767	769	775		1613	1618	1617									
rps12*	114	114	114	114					232	232	232	232	541	536	536	536	26	26	26	26
*clpP*	71	71	71	71	825	831	821	827	291	291	291	291	650	648	653	651	229	229	229	229
*ndhA*	553	554	553	553	1138	1111	1116	1137	539	541	539	539								
ndhB*	777	777	777		682	682	678		756	756	756									
*ycf3*	124	124	124	124	733	747	747	733	230	230	230	230	669	676	675	677	153	153	153	153
trnA-UGC*	38	38	38	38	813	803	813	803	35	35	35	35								
trnI-GAU*	42	42	42	42	945	945	945	945	35	35	35	35								
*trnL-UAA*	37	37	37	37	587	583	619	590	50	50	50	50								
*trnK-UUU*	37	37	37	37	2551	2560	2555	2563	28	29	29	29								
*trnV-UAC*	39				582				42											

*Euphorbia larica* = E.l, *Euphorbia smithii* = E.s, *Euphorbia esula* = E.e, *Euphorbia tirucalli* = E.t.

**Table 3:** Base composition of the *Euphorbia* species in the chloroplast genome.

	T/U				C				A				G				Length (bp)			
	E.l	E.s	E.e	E.t	E.l	E.s	E.e	E.t	E.l	E.s	E.e	E.t	E.l	E.s	E.e	E.t	E.l	E.s	E.e	E.t
**Genome**	32.7	32.5	32.7	32.6	18.1	18.1	18	18.1	31.7	31.7	31.8	31.8	17.5	17.6	17.6	17.5	162,258	162,172	160,512	163,091
**LSC**	34.5	34.3	34.5	34.3	16.7	16.8	16.6	16.7	32.8	32.7	32.9	32.9	16.0	16.1	16.1	16.1	91,537	91,158	90,309	91,259
**SSC**	35.0	34.6	34.8	35	15.7	15.8	15.9	15.8	34.9	34.9	35	34.9	14.3	14.7	14.3	14.3	18,239	18,603	17,023	1818
**IR**	28.9	29	28.7	29.1	20.5	20.5	21.9	20.4	28.6	28.5	29	28.6	22.0	22.1	20.4	21.9	26,291	26,206	26,590	26,832
**tRNA**	26.5	25.5	25.4	25.5	21.6	23.2	23.2	23.3	24.7	22.2	22.3	22.1	27.2	29.2	29.1	29.1	2887	2885	2925	2885
**rRNA**	18.8	18.8	18.8	18.8	23.7	23.7	23.7	23.7	25.8	25.8	25.7	25.7	31.8	31.8	31.8	31.8	2049	9050	9049	9049
**Protein Coding genes**	31.8	31.8	31.8	31.6	17.4	17.4	17.3	17.3	31.1	31.1	31.2	31.1	19.8	19.8	19.6	19.9	80,458	79,173	80,274	74,289
**1st position**	24.3	25.9	24.1	23.9	18.3	18.4	18.4	18.5	31.0	31.2	31.0	30.9	26.1	24.3	26.3	26.6	26,817	26,390	26,758	24,763
**2nd position**	32.6	32.7	28.4	38.4	19.9	19.8	19.9	19.9	29.7	29.8	29.8	29.9	17.5	17.5	17.4	17.5	26,817	26,390	26,758	24,763
**3rd position**	38.2	38.2	38.6	38.4	32.2	13.8	16.6	13.4	13.8	32.1	28.4	32.4	15.5	18.1	13.8	15.7	26,817	26,390	26,758	24,763

*Euphorbia larica* = E.l, *Euphorbia smithii* = E.s, *Euphorbia esula* = E.e, *Euphorbia tirucalli* = E.t.

## Supplementary Materials

The following are available online at https://www.mdpi.com/2223-7747/9/2/199/s1, Figure S1: Frequency of identified SSR motifs in different repeat class types, Table S1: Genes in the sequenced *E. smithii* and *E. larica* chloroplast genomes.
