# Supplementary figures and images for: Comparative Chloroplast Genomics of Endangered Euphorbia Species: Insights into Hotspot Divergence, Repetitive Sequence Variation, and Phylogeny
Source: Plants (Basel). 2020 Feb 5;9(2):199. doi: 10.3390/plants9020199 (PMC7076480; doi:10.3390/plants9020199)

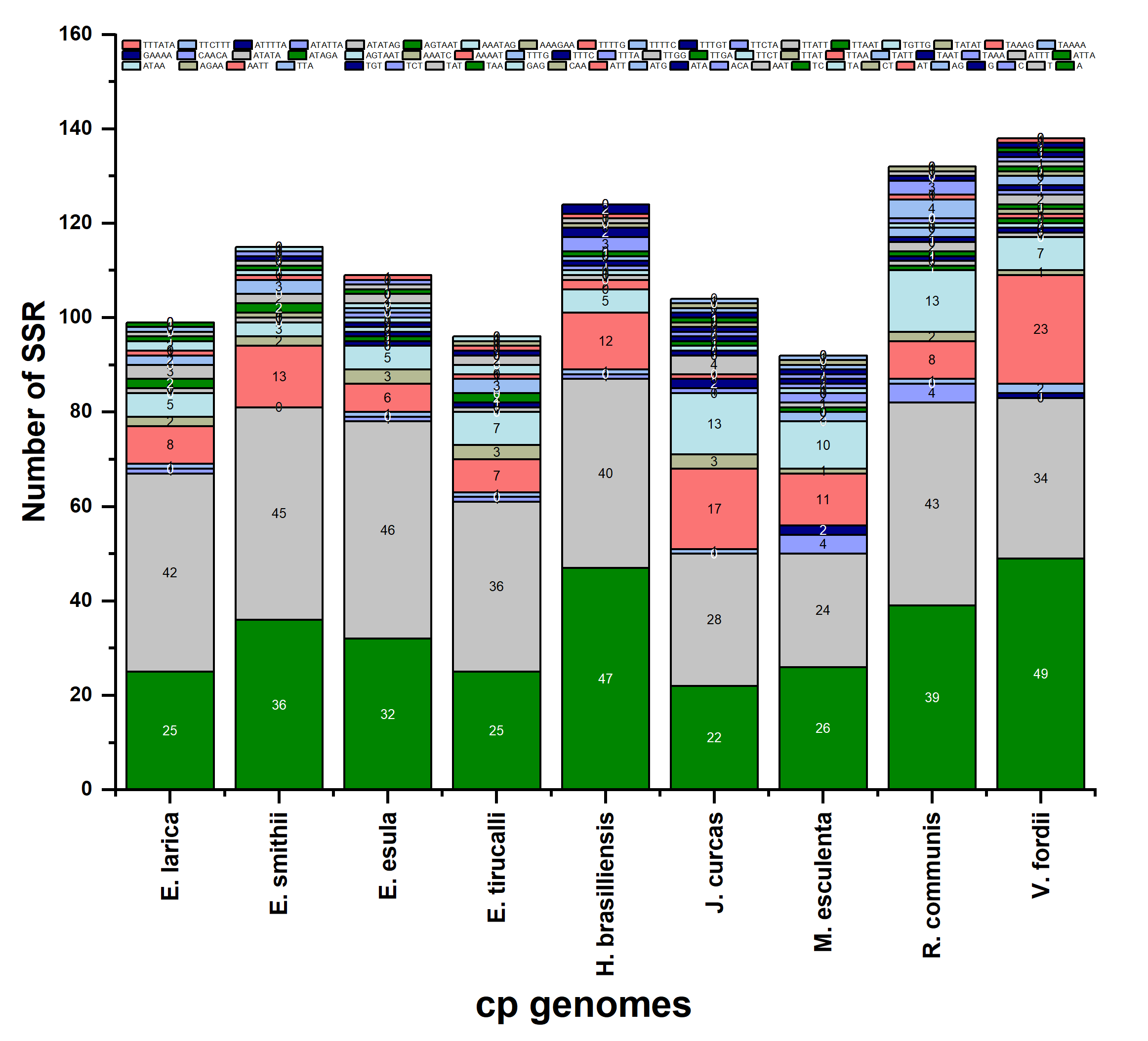

Supplement: Supplementary file 1 [file plants-09-00199-s001.zip › Figue S1.tif]
