# Supplementary material for: Comparative Chloroplast Genomics of Endangered Euphorbia Species: Insights into Hotspot Divergence, Repetitive Sequence Variation, and Phylogeny
Source: Plants (Basel). 2020 Feb 5;9(2):199. doi: 10.3390/plants9020199 (PMC7076480; doi:10.3390/plants9020199)
Supplement: Supplementary file 1 [file plants-09-00199-s001.zip › Table S1.doc]

**Table S1. Genes in the sequenced *Euphorbia* *E. smithii* and *E. larica* chloroplast genomes.**

| **Category** | **Group of genes** | **Name of genes** |
| --- | --- | --- |
| **Self-replication** | Large subunit of ribosomal proteins | *rpl2*, *14*, *16*, *20*, *23*, *32*, *33*, *36* |
|  | Small subunit of ribosomal proteins | *rps2, 3, 4, 7, 8, 11, 14,15 ,18, 19* |
|  | DNA dependent RNA polymerase | *rpoA, B, C1, C2* |
|  | rRNA genes | *rrn 4.5, rrn 5, rrn 16, rrn23* |
|  | tRNA genes | *trnA-UGC, trnC-GCA, trnD-GUC, trnE-UUC trnF-GAA, trnfM-CAU, trnG-UCC, trnH-GUG, trnI-CAU, trnI-GAU, trnK-UUU, trnL-CAA, trnL-UAA, trnL-UAG, trnM-CAU, trnN-GUU, trnP-GGG, trnP-UGG, trnQ-UUG, trnR-ACG, trnR-UCU, trnS-GCU, trnS-GGA, trnS-UGA, trnT-GGU, trnT-UGU, trnV-GAC, trnV-UAC, trnW-CCA, trnY-GUA* |
| **Photosynthesis** | Photosystem I | *psaA, B, C, I, J,* |
|  | Photosystem II | *psbA, B, C, D, E, F, H, I, J, K, L, M, N, T, Z* |
|  | Cytochrome b6/f complex | *petA, B, D, G, L, N* |
|  | ATP synthase | *atpA, B, E, F, H, I* |
|  | Rubisco | *rbcL* |
|  | Maturase | *matK* |
|  | Protease | *clpP* |
|  | Envelop membrane protein | *cemA* |
|  | Subunit Acetyl- CoA-Carboxylate | *accD* |
|  | c-type cytochrome synthesis gene | *ccsA* |
| **Unknown** | Conserved Open reading frames | *Ycf1, 2, 3, 4, 15* |
